# Structural studies of the phosphoribosyltransferase involved in cobamide biosynthesis in methanogenic archaea and cyanobacteria

**DOI:** 10.1038/s41598-022-21765-5

**Published:** 2022-10-13

**Authors:** Victoria L. Jeter, Anne H. Schwarzwalder, Ivan Rayment, Jorge C. Escalante-Semerena

**Affiliations:** 1 Department of Microbiology, University of Georgia, Athens, GA 30602 USA; 2 Department of Biochemistry, University of Wisconsin, Madison, WI 53706 USA

**Keywords:** Microbiology, Structural biology

## Abstract

Cobamides (Cbas) are coenzymes used by cells across all domains of life, but de novo synthesis is only found in some bacteria and archaea. Five enzymes assemble the nucleotide loop in the alpha phase of the corrin ring. Condensation of the activated ring and nucleobase yields adenosyl-Cba 5′-phosphate, which upon dephosphorylation yields the biologically active coenzyme (AdoCba). Base activation is catalyzed by a phosphoribosyltransferase (PRTase). The structure of the *Salmonella enterica* PRTase enzyme (i.e.,* Se*CobT) is well-characterized, but archaeal PRTases are not. To gain insights into the mechanism of base activation by the PRTase from *Methanocaldococcus jannaschii* (*Mj*CobT), we solved crystal structures of the enzyme in complex with substrate and products. We determined several structures: (i) a 2.2 Å structure of *Mj*CobT in the absence of ligand (apo), (ii) structures of *Mj*CobT bound to nicotinate mononucleotide (NaMN) and α-ribazole 5′-phosphate (α-RP) or α-adenylyl-5′-phosphate (α-AMP) at 2.3 and 1.4 Å, respectively. In *Mj*CobT the general base that triggers the reaction is an aspartate residue (Asp 52) rather than a glutamate residue (E317) as in *Se*CobT. Notably, the dimer interface in *Mj*CobT is completely different from that observed in *Se*CobT. Finally, entry PDB 3L0Z does not reflect the correct structure of *Mj*CobT.

## Introduction

Cobamides (Cbas) are cobalt-containing cyclic tetrapyrroles belonging to a family of cofactors called ‘the pigments of life’. In addition to Cbas, chlorophylls, hemes, and coenzyme F_430_ are other metal-containing cofactors that make up this family of biologically important compounds^1^. De novo Cba biosynthesis is restricted to some bacteria and archaea, yet Cba-dependent enzymes are found in cells from all domains of life^2,3^. The prevalence of Cba-dependent reactions has implicated Cbas as an important compound in communities of organisms^4–6^. Cbas are utilized in a diverse array of chemical reactions including enzyme catalyzed carbon skeleton rearrangements, methyl-group transfers and reductive dehalogenation. Additionally, Cbas have been shown to act as photoreceptors in the regulation of carotenoid biosynthesis^7^.

**Figure 1:**
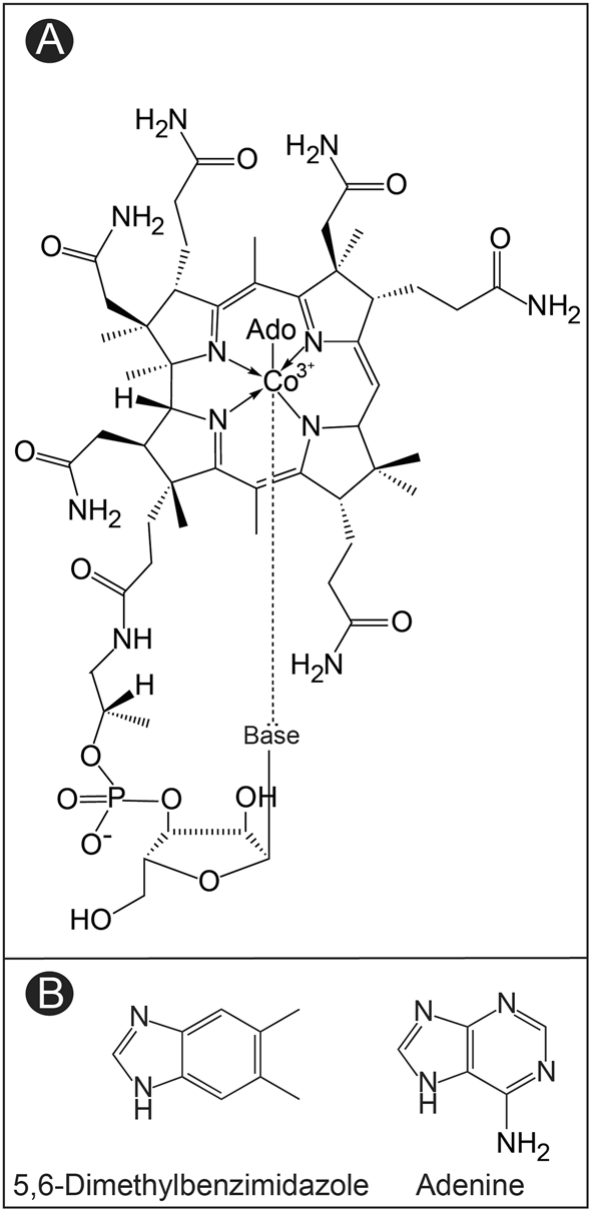
Structure of adenosylcobalamin (AdoCbl), and pseudo-adenosylcobalamin (psAdoCbl). (**A**) General structure of an adenosylcobamide, where ‘Base’ refers to diverse nitrogenous nucleobases found in cobamides. Only nitrogenous nucleobases can form a coordination bond (dashed line) with the cobalt ion of the tetrapyrrole ring. In some cases, the nucleobase can be either phenol or *p-*cresol, which cannot coordinate with the cobalt ion. (**B**) 5,6-Dimethylbenzimidazole (DMB) is the nucleobase found in cobalamin, while adenine (Ade) is the nucleobase found in pseudocobalamin (psCbl).

Cbas are structurally unique from other metal-containing cofactors in that they possess upper (*Coβ*) and lower (*Coα*) axial ligands (Fig. 1). Cobalamin (Cbl, B_12_) is defined by a 5,6-dimethylbenzimidazole (DMB) lower ligand nucleobase. Adenosylcobalamin (AdoCbl), the coenzyme form of Cbl, is characterized by an upper 5′-deoxyadenosyl moiety which participates in radical chemistry reactions. The lower nucleobase is a source of diversity among Cba structures, incorporating purines, benzimidazoles, and in some cases phenolics^8,9^. The Cba that contains adenine as the nucleobase is known as pseudo-cobalamin (psCbl), and it is relevant to this work because psCbl has been reported to be synthesized by methanogenic archaea, and adenine has been shown to be a substrate for *Mj*CobT^10,11^.

De novo synthesis of Cbas can be divided into early and late steps. The early steps are responsible for the assembly of the corrin ring, while the late steps are responsible for activating and attaching the corrin ring and the nucleobase.

**Figure 2:**
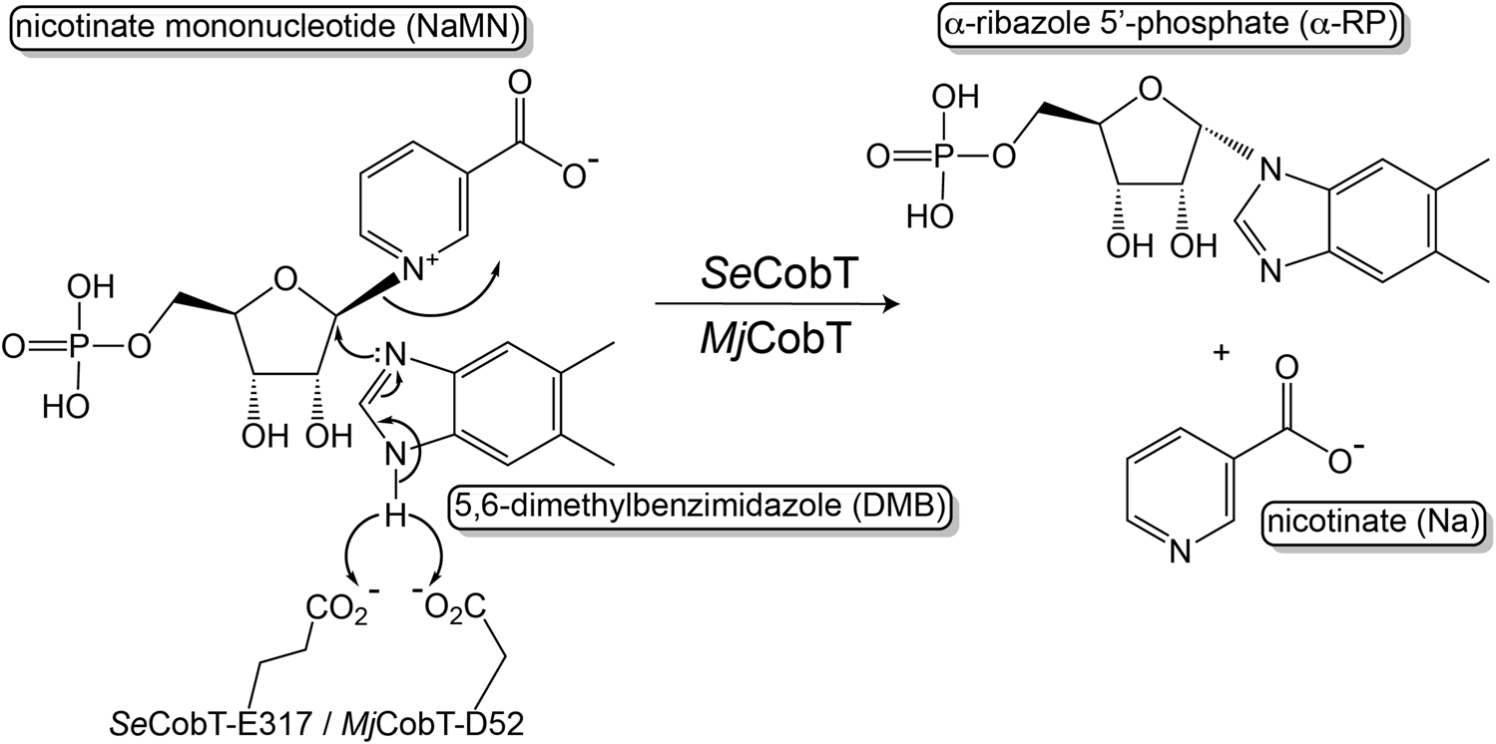
Activation of DMB into its alpha ribotide, α-ribazole-5′-phosphate (α-RP). In this reaction mechanism, residues E317 of *Se*CobT and D52 of *Mj*CobT act as general bases to trigger the attack by an unpaired set of electrons of one of the imidazole nitrogens on the alpha face of the molecule resulting in a release of nicotinate and the formation of α-RP, a *bona fide* intermediate of the nucleotide loop assembly pathway^12,13^.

The late steps are also referred to as the Nucleotide Loop Assembly (NLA) pathway. In *S. enterica*, the nicotinate mononucleotide (NaMN):base phosphoribosyltransferase (*Se*CobT, EC 2.4.2.21) catalyzes the activation of nucleotide bases by synthesizing α-riboside monophosphates α-ribotides). The canonical CobT reaction and its proposed mechanism are shown in Fig. 2.

In the case of AdoCbl biosynthesis, *Se*CobT generates α-ribazole 5′-phosphate (α-RP) from DMB. In vitro, *Se*CobT exhibits broad substrate specificity and has been shown to act on a diverse range of purine and benzimidazole analogues^14–16^.

One of the hallmarks of base activation by *Se*CobT is the generation of a unique α-*N*-glycosidic linkage between C1 of ribose and N1 of imidazole (Fig. 2). Numerous structures of *Se*CobT have been solved, based on which a general-base catalysis mechanism using a conserved glutamate has been proposed. Such a mechanism is supported by mutational and biochemical studies, which established our current understanding of the active site of *Se*CobT^14–18^ (Fig. 2).

The enzymes that assemble the nucleotide loop in methanogenic archaea have not been studied in detail in a single organism. Our group has reported several studies in *Methanopyrus kandleri, Methanosarcina mazei* Gö1, and *Methanobacterium thermoautotrophicum* ∆H^19–21^. However, most of our work with methanogenic archaea has been performed with *Methanocaldococcus jannaschii.* In this archaeon, detailed biochemical and mutational/functional analyses and NMR and crystal structures of the GTP:adenosylcobinamide-phosphate guanylyltransferase (a.k.a. *Mj*CobY) enzyme have been reported^22–24^. The *Mj*CobY enzyme activates the corrin ring intermediate prior to its condensation with α-RP, a reaction that yields AdoCba-5′-P, which is dephosphorylated to yield the final product of the pathway, i.e., AdoCba.

Recently, we performed the biochemical characterization of the *Mj*CobT enzyme, and based on this work we proposed that the available crystal structure of *Mj*CobT (PDB 3L0Z) was probably incorrect^11^, triggering the work reported herein. Notably, the PDB 3L0Z structure provided limited mechanistic information because it was devoid of substrates or products, and to date, the experimental details of how the structure was obtained have not been published. In addition, no functional characterization of the *Mj*CobT enzyme linked to PDB 3L0Z has been reported.

Importantly, our work showed that *Mj*CobT can activate 5-hydroxybenzimidazole (5-HO-Bza, a.k.a. Factor III) and adenine^11^, two nucleobases that are commonly found in Cbas synthesized by methanogenic archaea^10^.

The work reported here provides structural and mechanistic insights about the *Mj*CobT enzyme. We report crystal structures of *Mj*CobT without ligands (apo form) and in complex with NaMN substrate with the α-RP and α-AMP products determined to a 2.2, 2.3, and 1.4 Å resolution, respectively. We confirmed our predictions that the biologically active *Mj*CobT is a dimer, not a trimer, as shown in PDB 3L0Z. Additionally, we show significant differences in the dimeric interface compared to that of *Se*CobT. Though the overall protein fold is comparable between *Se*CobT and *Mj*CobT, we see a striking difference in the residue composition and location within the active site, and identified an aspartate residue as the putative general base that we propose triggers the formation of α-ribotides that can be used as substrates to make Cbas in this archaeon (Fig. 2).

## Results and discussion

Three structures of *Mj*CobT are reported here. First an apo structure that includes no ligands in its active site, second, a complex of *Mj*CobT with NaMN and nicotinic acid with α-RP, and third, *Mj*CobT in complex with α-adenosyl 5′-phosphate (α-AMP) and nicotinic acid. These structures were determined to 2.2, 2.3, and 1.4 Å respectively. Each of these structures provide unique insights into the assembly, ligand binding, specificity, and active site determinants of *Mj*CobT as described below. None of the structures described here contain a free base, although they were present in all crystallization experiments. This is true for the apo structure, which contains no ligands in the active site.

### *Mj*CobT oligomerization state

**Figure 3:**
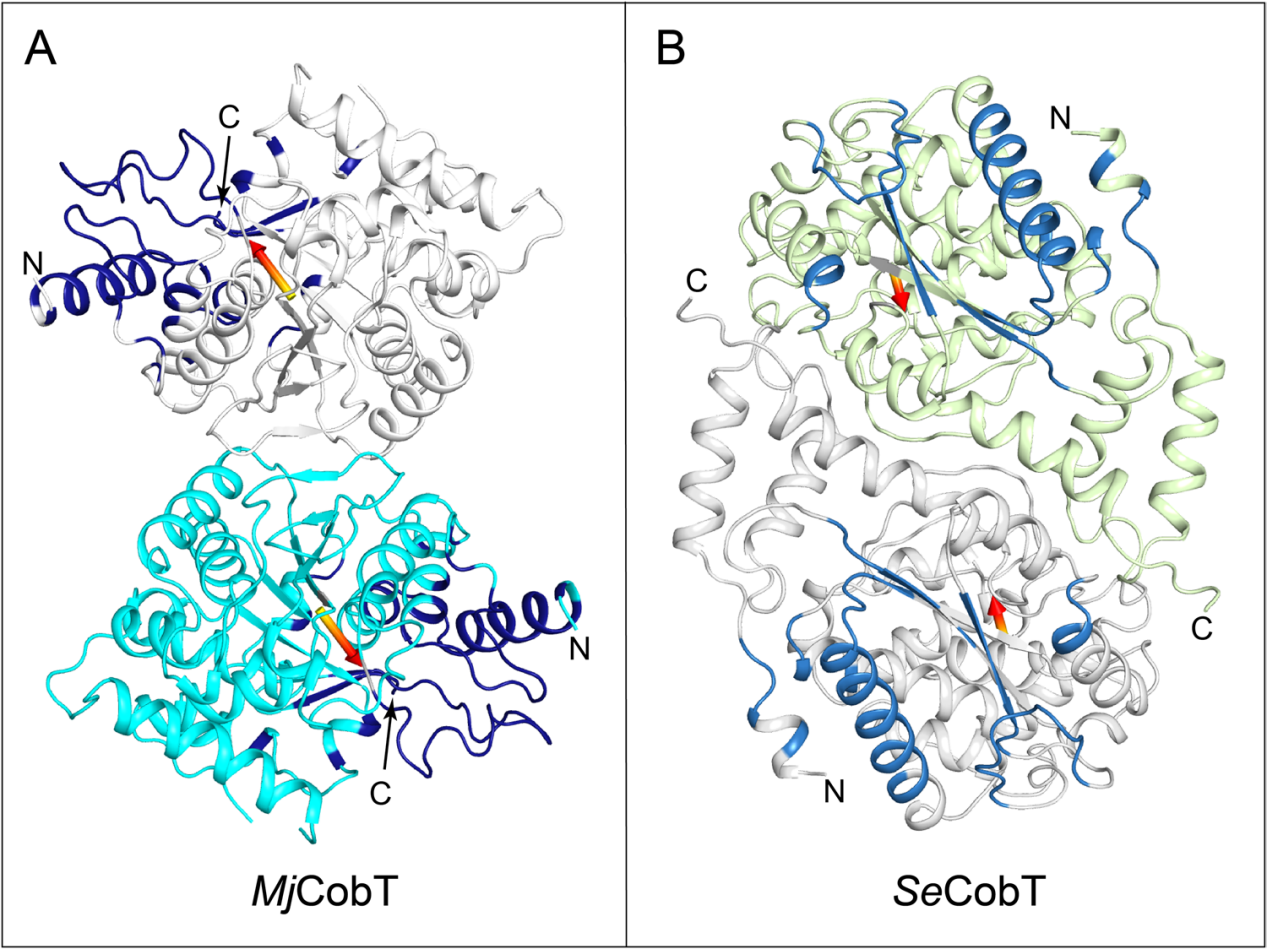
Comparison of the quaternary structures of *Mj*CobT and *Se*CobT. The orientation of *Mj*CobT in its dimer relative to that seen in *Se*CobT (1L4B)^17^ is completely different as indicated by the arrows that depict a similar vector in each monomer. (**A**) shows the quaternary of apo-*Mj*CobT. The equivalent residues that form the dimer interface in *Se*CobT are depicted in dark blue. Conversely, the residues in *Se*CobT that correspond to the interface in *Mj*CobT are depicted in blue in (**B**). This dramatic difference in assembly follows the rms differences between *Mj*CobT and *Se*CobT which are 2.2 Å for 248 equivalent alpha-carbon atoms. This reveals that the enzymes contain a similar fold but differ in their exterior loops.

Previous structural studies of apo *Mj*CobT suggested that the enzyme might assemble as a trimer since this arrangement was observed in the crystal lattice (RCSB PDB 3L0Z). In contrast, isothermal titration calorimetry and analytical ultracentrifugation analysis show that the biologically active form of the enzyme is a dimer in solution^11^. All of the structures reported here are consistent with a dimeric assembly and hence are in agreement with the biophysical studies. A ribbon representation of the apo dimer described here is shown in Fig. 3A.

The supposition that *Mj*CobT was a trimer arose because the protein crystallized with three protomers per asymmetric unit arranged with three-fold symmetry. However, the presence of a crystallographic two-fold generates a molecule with P32 symmetry in which the dimers assemble with the same relationship seen in the structures reported here. Indeed, the rms difference between dimers in the earlier and current apo structure is 0.86 Å for 662 structurally equivalent residues^25^. The dimeric packing arrangement present in the original structure (3L0Z) was described earlier in the paper by Jeter et al.^11^ and is confirmed here. The total buried surface area per protomer for this dimer is 945 Å^2^ where 70% of this is hydrophobic^26^. Interestingly, the total buried surface area for the trimer seen in the original structure (3L0Z) is only 515 Å^2^ per subunit, which is significantly less than observed in the biological dimeric assembly.

The mode of dimerization observed in *Mj*CobT is markedly different from that seen in *Se*CobT. As opposed to *Mj*CobT, *Se*CobT forms a tight dimer with an extensive interface (Fig. 3B). In this case the buried surface area is 1720 Å^2^ per protomer. As can be seen, the components that constitute the interfaces are unrelated and lie on approximately opposite sides of each homologue. This has implications for the active site, since the binding pocket for the base in *Se*CobT is formed by components of both polypeptide chains whereas in *Mj*CobT the ligand binding sites are formed from a single polypeptide chain, as described below.

### Structure of *Mj*CobT bound to α-RP and NaMN

*Mj*CobT was crystallized in the presence of DMB at pH 7.0 and gave a crystal lattice that contained two protein subunits per asymmetric units. These crystals were soaked in 1 mM NaMN and a nucleotide base overnight prior to freezing. During that time the enzyme catalyzed a transferase reaction. The contents of the two active sites are different. One subunit contains NaMN, whereas the second includes α-RP and nicotinic acid. This provides an opportunity to observe substrate and product in the same crystal lattice. The polypeptide chain for both subunits extends continuously from Met 1 to Tyr 348 except for a short break between Pro 68 and Gly 73 where the structure was disordered. The two subunits are very similar as measured by a rms difference of 0.253 Å for 334 equivalent α-carbon atoms.

**Figure 4:**
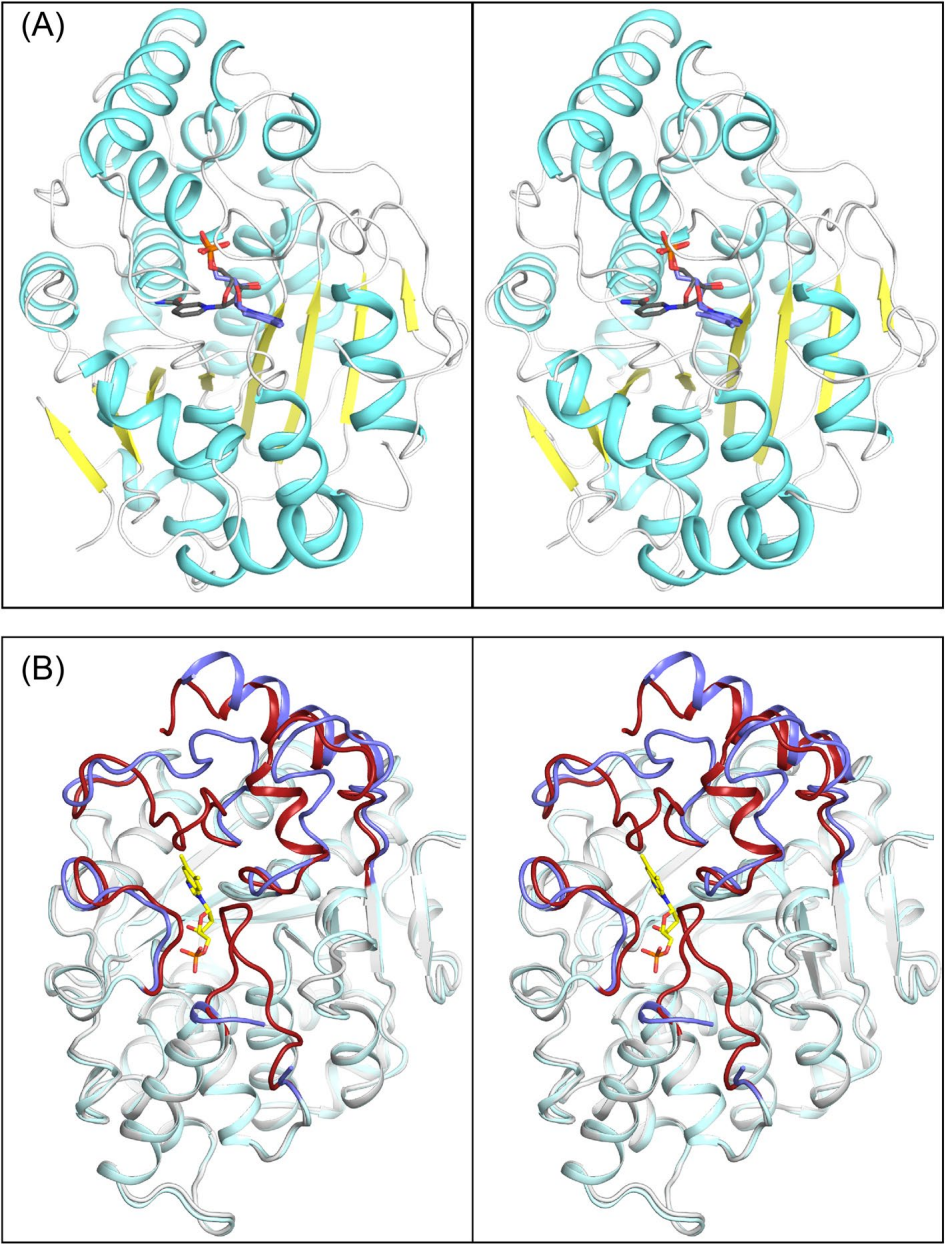
Stereo view of the location of the active site in *Mj*CobT and the conformational changes that occur on ligand binding. (**A**) shows a superposition of NaMN and α-RP from crystalline complex of substrate and products and reveals the entirety of the active site located at the C-terminal ends of the β-sheet. (**B**) shows a comparison between the apo-structure and subunit B that contains α-RP. The loops that show significant change between the apo *Mj*CobT and the complex with α-RP and nicotinic acid are depicted in blue and dark red respectively. This reveals that the active site is open to solvent in the apo structure, but is enclosed when product is bound. These changes in tertiary structure are associated primarily with those regions of the protein that constitute the binding site for the nitrogenous base.

A superposition of the active site that contains α-RP and nicotinic acid on that which carries NaMN is shown in Fig. 4A.

This reveals the extent of the substrate binding pocket. The protein fold consists of an eight-stranded mostly parallel β-sheet with connecting α-helices on both sides where the substrate binding pocket is built from the connecting loops and resides at the end of the *C*-terminal end of β-strands. It is a compact globular protein in which the active site encloses the substrates and products. Access to the active site is most likely provided by movement of the outermost loops as indicated by a comparison of the apo structure with the polypeptide chain that carries α-RP shown in Fig. 4B. Superposition of these two structures shows that significant changes occur in the loops extending from Ile 37 to Ile 45, Ser 293 to Lys 314, and Pro 333 to Tyr 348, such that the active site of the apo structure is open to solvent in contrast to that of the complex with either NaMN or α-RP and nicotinic acid. The rms difference between these two structures is 0.94 Å for 329 equivalent residues, indicating the significant conformational changes that occur when substrates bind. Strikingly, residues Lys 173 to Asn 181 that were disordered in the apo structure became ordered upon ligand binding and contribute to the distal component of the binding pocket for the nitrogenous base. Altogether, the movement of the surface loops serves to close the active site on substrate binding.

The electron density for NaMN and α-RP and nicotinic acid is unequivocal, although the occupancy of α-RP is somewhat less than 1.0 (Fig. 4). NaMN lies at the base of the active site for chain A where there are extensive hydrogen bonding interactions with the phosphoryl moiety, and specific interactions with the ribose 2′ and 3′-oxygens and the carboxylate group of NaMN (Fig. 4A). The phosphoryl oxygens participate in hydrogen bonds to the amide hydrogen and Oγ of Thr 156, the Oγ of Ser 38 and amide hydrogen of Gly 154, and the amide hydrogen Gly 177. The carboxylate of NaMN is coordinated to the amide hydrogen and Oε of Gln 241 and the amide hydrogen of Thr 240. The ribose O3′ of NaMN is coordinated to the amide hydrogen of Val 152. The C1′ of the ribose faces out into a cavity that lies closer to the surface loops than NaMN. This cavity forms the binding pocket for the nitrogenous base as revealed by the complex of α-RP and nicotinic acid observed associated with chain B (Fig. 4B).

The coordination of the ribose, phosphate, and nicotinic acid moieties in the active site that complexed α-RP is very similar to what was observed for NaMN. However, there are additional hydrogen bonds between O2’ of the ribose and Oγ1 and Oγ2 of Glu 150. The binding site for DMB is formed primarily by hydrophobic components of three surface loops that extend from Val 40–Thr48, Pro 68–Pro 77, and Gly 311–Val 313. There is a hydrogen bond between an imidazole nitrogen of DMB and Oγ of Asp 52 that fulfills the hydrogen bonding potential of the benzimidazole ring (Fig. 4B). As noted earlier, there is a disordered section in the polypeptide chain that extends from Ile 69 to Thr 72. This short section of polypeptide chain lies at the distal end of the nitrogenous base and is disordered and cannot be modelled in either active site in the complex with NaMN and α-RP and nicotinic acid, although it is ordered in the presence of the α-AMP and in the apo structure. The position of the nitrogenous base in α-RP suggests that NaMN binds first in the active site, though this can only be confirmed by detailed kinetic studies.

### Accommodation of alternative nitrogenous bases

**Figure 5:**
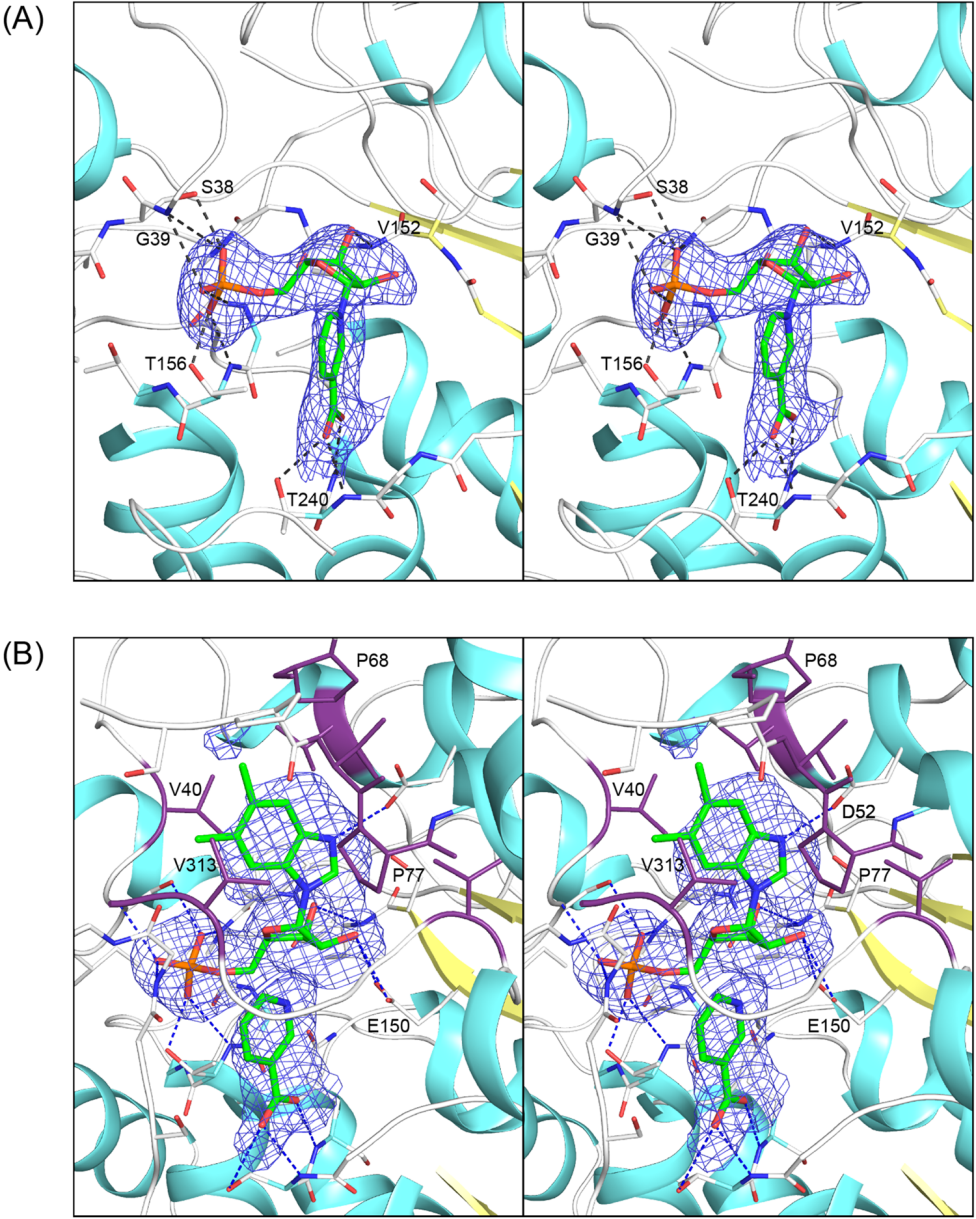
Stereo electron density for NaMN and α-RP and nicotinic acid and their hydrogen bonding interactions. Shown in stereo in (**A**) and (**B**) are the observed electron densities for NaMN and α-RP and nicotinic acid, respectively. The omit maps were calculated with coefficients of the form *F*_o_ − *F*_c_ where *F*_o_ and *F*_c_ were the native and calculated structure factor amplitudes, respectively. The map was contoured at 2.5σ. (**A**) shows the extensive hydrogen bonding interactions of the nicotinamide ring, ribose sugar, and phosphate moiety. The same interactions are observed for these moieties in the active site that carries α-RP and nicotinic acid, together with the interactions with DMB. (**B**) shows the side chains that surround the nitrogenous base which are mostly hydrophobic, however there is a hydrogen bond between N3 of DMB and Oγ of Asp 52.

The structure of the complex with α-AMP was determined to 1.4 Å resolution. This structure was determined to understand the broad specificity of *Mj*CobT ^11^. As noted earlier, the reaction product was prepared in situ by soaking the crystals in NaMN and adenine. In this case the crystals were highly ordered and the position of α-AMP is unequivocal (Fig. 5A).

The overall position of the adenine α-ribotide is exceedingly similar to that α-RP so that the primary question is how the enzyme can accommodate nitrogenous bases that differ in both the composition of the aromatic ring and its side chain substitutions (Fig. 5B). Superposition of the two structures demonstrates that this is accomplished in this instance by conformational changes in the polypeptide chain between Ile 69–Thr 74. As noted above, this section is ordered with α-AMP and disordered with α-RP. A change in the conformation of this loop, when α-RP is bound, is necessary because the two methyl groups in DMB overlap with the ordered loop seen in the complex with α-ribotide. It is conceivable that the disordered section allows for varied substrate specificity which is a hallmark of CobT.

**Figure 6:**
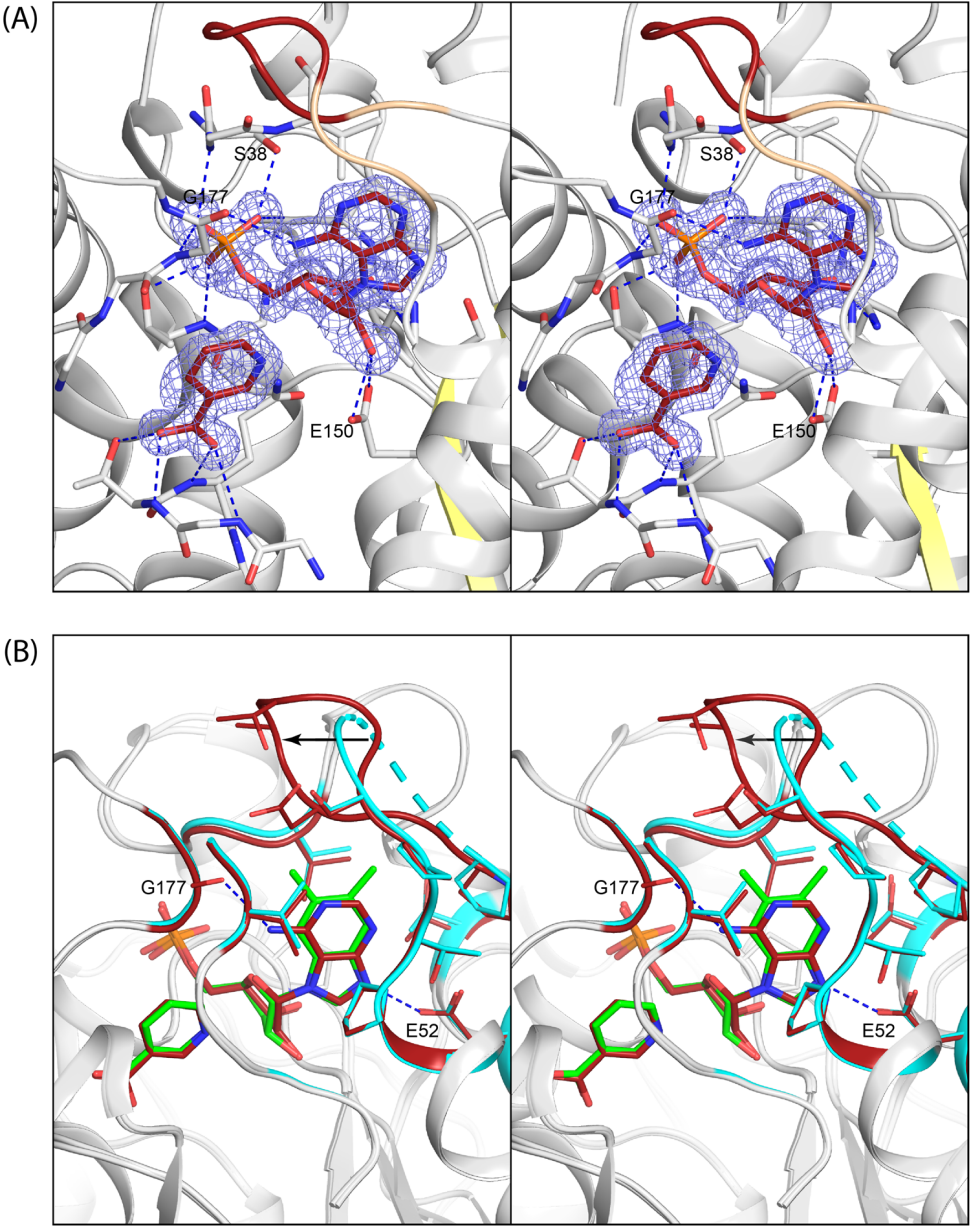
Stereo electron density and model for the α-AMP and its similarity to α-RP. Shown in stereo in (**A**) are the observed electron density for α-AMP and nicotinic acid. It also shows the base specific interaction between the carbonyl oxygen of Gly 177 and the amino group of adenine. Note again that there are additional hydrogen bonds between O2′ of the ribose and Oγ1 and Oγ2 of Glu 250 relative to the complex with NaMN. (**B**) shows the close similarity in the mode of binding of adenine ribotide and α-RP. The major difference is ordering of the loop between Ile 69–Thr 72 such that it encloses the purine ring.

The remainder of the side chains that interface the nitrogenous bases adopt essentially the same conformation (Fig. 6). An adenine specific interaction arises between the carbonyl oxygen of Gly 177 and the amino group of adenine, but this does not alter the conformation of the active site. Interestingly, there are no polar interactions between the pyrimidine ring nitrogen N1 and N3 of the purine, which instead lie at van der Waals distance from the surrounding components of the binding pocket. Note again, the additional hydrogen bonds between O2’ of the ribose and Oγ1 and Oγ2 of Glu 550 relative to the complex with NaMN, suggesting that this is a feature of the ribotide product complex.

### Comparison with *S. enterica* CobT

**Figure 7:**
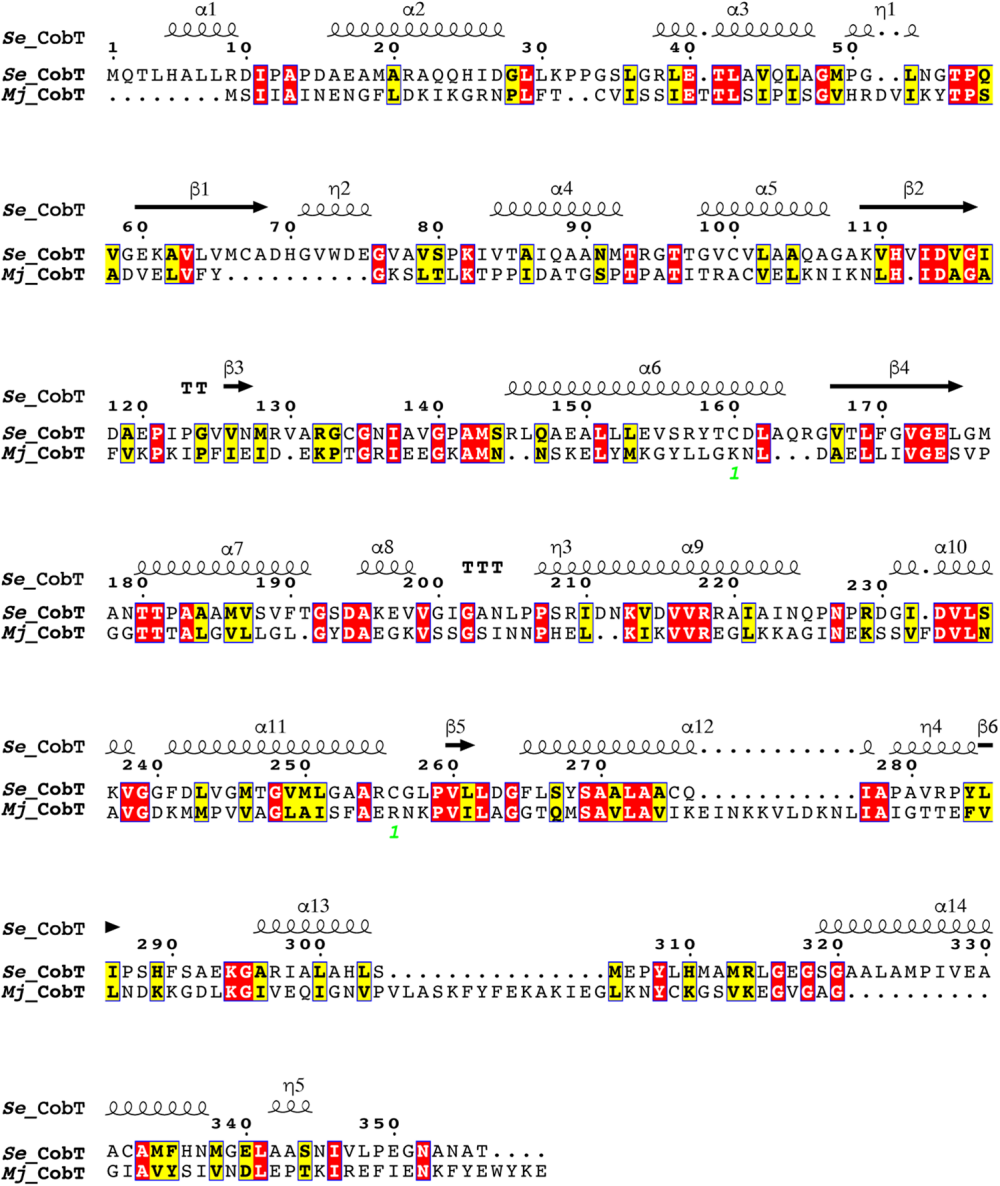
Comparison of the *Mj*CobT and *Se*CobT primary sequences. This alignment was constructed using the online ESPrit 3 algorithm^27^.

As shown in Fig. 7, the primary sequences of *Se*CobT and *Mj*CobT are only 18% identical and 32% similar.

**Figure 8:**
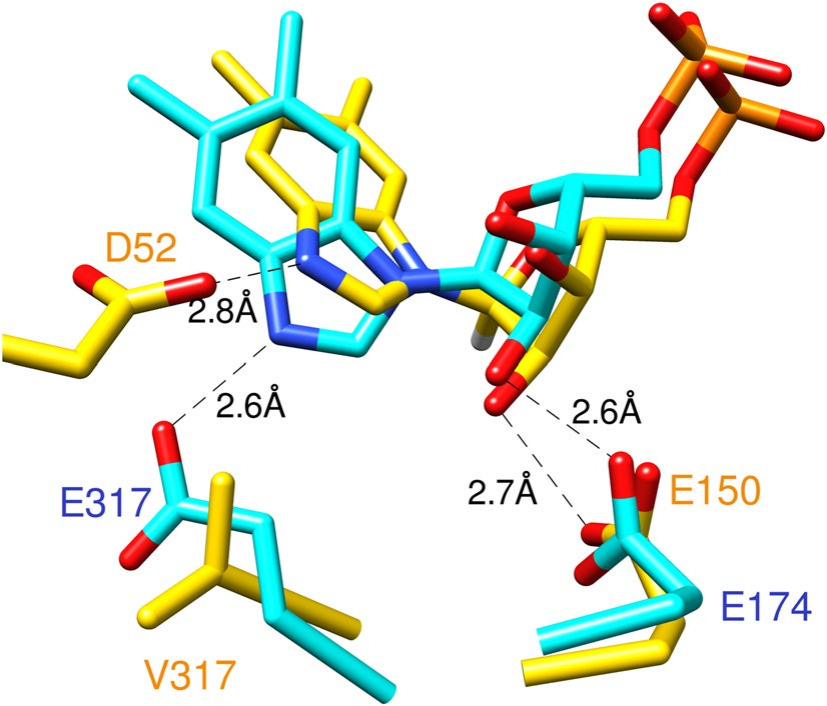
Active Site overlay of *Mj*CobT and *Se*CobT. In this figure, α-RP, the product of the *Se*CobT and *Mj*CobT reactions is shown in cyan and yellow, respectively. As seen in the figure, in the active site of *Mj*CobT, α-RP is held by interactions between the N3 atom of the imidazole ring with residue D52. Very similar interactions are observed between the N3 atom of α-RP and residue E317 of the active site of *Se*CobT. In both active sites α-RP is further held in position by interaction between the 2’ hydroxyl group of α-RP and either E150 (in the case of *Mj*CobT) or E174 (in the case of *Se*CobT). As shown in the figure, in *Mj*CobT, position 317 is occupied by a valine residue. Position 52 in *Se*CobT is occupied by a glycine residue. This image was generated using PyMOL 2.5 (Schrödinger).

However, the overall protein fold of *Mj*CobT is fundamentally the same as that of *Se*CobT where the rms difference between 248 equivalent α-carbon atoms is 2.2 Å. There is also a well-established equivalency in their enzymatic function, specificity and evolutionary relationship^11^. As such, it would be anticipated that the active site residues would be found at equivalent locations in their sequences. However, close examination of the active sites reveals a major difference in the identity and position in the sequence of the active site base. In *Se*CobT, the active site base is Glu 317^18^. This functional group is required since the imidazole moiety of the aromatic base will carry a single proton on one of the ring nitrogen atoms at pH of ~ 7. The first pKa of benzimidazole is 5.4 whereas the second pKa is ~ 11.5. It is the latter pKa that is important in the chemical reaction (Fig. 2). In *Mj*CobT the structurally equivalent amino acid is Val 317, where the side chains of Glu and Val 317 adopt similar positions. Instead, Asp 52 is the base in *Mj*CobT as revealed in Fig. 8. The use of an alternative base is possible because the benzimidazole ring in *Mj*CobT is rotated approximately 30° relative to that observed in *Se*CobT. This places the protonated nitrogen opposite the side chain of Asp 52.

## Concluding remarks

This study reports the corrected structure of *Mj*CobT, the only archaeal CobT to be functionally characterized. Additionally, structures containing both substrate and product in the active site were dissected. This work elucidated the structural differences between *Mj*CobT and the extensively characterized *Se*CobT. The overall protein folds of these two enzymes are similar, yet the specifics of active site construction and dimer interactions are unique. The conservation of active sites that can accommodate a broad range of substrates indicate a strong selection for maintaining the ability to synthesize diverse Cbas in the drastically different environments occupied by *S. enterica* (e.g., the gut of poultry, reptiles, humans; mesophilic range temperatures) *versus* the marine hydrothermal vents inhabited by *M. jannaschii* and other extreme thermophiles.

## Methods

### Cloning of the gene encoding *Mj*CobT

*Mj*CobT was cloned into vector backbone pTEV5 as previously reported^11^.

### Protein expression and purification

The plasmid carrying *Mj*CobT was used to transform BL21-CodonPlus *E. coli* cells (Agilent Technologies). Cultures were grown in LB medium with kanamycin (50 µg/L) and chloramphenicol (50 µg/L) until an optical density of A_600_ = 0.8 was reached. Cells were chilled on ice before inducing protein expression by addition of isopropyl β-d-1-thiogalactopyranoside (IPTG, 1 mM) for a 24 h growth at 16 °C. The cells were harvested by centrifugation at 4˚C, washed in 2-[4-(2-hydroxyethyl)piperazin-1-yl]ethanesulfonic acid (HEPES) buffer (20 mM, pH 7.6) containing NaCl (100 mM), and centrifuged again before storing at − 80 °C until used.

All protein purifications were carried out at 4 °C. In preparation for purification, cells were lysed by sonication on ice in lysis buffer (25 mM Tris–HCl pH 8.0, 50 mM NaCl, 20 mM imidazole) with lysozyme (1 mg/mL), DNaseI (25 mg/mL), and protease inhibitor phenylmethylsulfonyl fluoride (PMSF) 0.5 mM added to the cells. The lysate was incubated at 80 °C for 30 min to precipitate native *E. coli* proteins before clarification at 40,000×*g* at 21 °C for 30 min in an Optima LE-80 K Ultracentrifuge (Beckman) using a Ti-45 rotor (Beckman/Coulter) that was pre-cooled 4 °C prior to use. Clarified lysate was loaded over a 5-mL Ni–NTA column, washed with ten column volumes (CV) of wash buffer (25 mM Tris–HCl pH 8.0, 500 mM NaCl, 20 mM imidazole) and six CVs of a second wash buffer with imidazole increased to 40 mM, before eluting with six CVs of Tris–HCl buffer (25 mM, pH 8.0) containing NaCl (500 mM), and imidazole (500 mM). Recombinant tobacco etch virus protease protease with a non-cleavable hexahistidine tag fused to its N terminus (H_6_-rTEV) was purified as described elsewhere^28^ was mixed with eluted *Mj*CobT to cleave the hexahistidine tag fused to the N terminus of the protein, and was allowed to react overnight at 21 °C in Tris–HCl buffer (25 mM, pH 8.0) containing and NaCl (500 mM). After confirming cleavage by SDS-PAGE^29^, the protein was passed over a Ni–NTA column to separate the H_6_-rTEV protease from purified *Mj*CobT*.* Purified *Mj*CobT was then dialyzed into a Tris–HCl buffer (25 mM, pH 8.0) containing NaCl (250 mM) overnight at 21 °C. Before crystallization, *Mj*CobT was concentrated to 13.3 mg/mL by centrifugation at 4000×*g* in a 30 K molecular weight cut-off Amicon Ultra Centrifugal Filter, and flash-frozen as 30 mL droplets in liquid nitrogen for storage at – 80 °C.

### Crystallization

The purified *Mj*CobT protein (13.3 mg/mL) was screened for crystal growth at both room temperature and 4˚C across a 144-condition hanging drop, sparse-matrix screen developed by the Rayment Laboratory. The enzyme was examined for crystal growth in its apo-state, or in the presence of substrates. Drops were seeded after 24 h to incite crystal growth. All crystals were obtained at room temperature. X-ray diffraction quality crystals were transferred to a cryogenic solution containing 20% (v/v) glycerol or ethylene glycol and supplemented with additional substrates to enhance ligand binding within the crystal matrix. Crystals were allowed to soak in these conditions overnight before flash freezing for storage before data collection.

Apo crystals were obtained in 100 mM 2-(*N*-morpholino)ethanesulfonic acid (MES)/acetate buffer pH 5.5 with 8.4% polyethylene glycol (PEG) 8 K and 1 mM 5-hydroxybenzimidazole (5-OH) added to the protein before screening. X-ray diffraction quality crystals were then soaked in a solution containing 20% ethylene glycol, 100 mM (MES)/acetate buffer pH 5.5, 16% PEG 4 K, and 5 mM 5,6-dimethylbenzimidazole (DMB), though neither of the substrates (5-OH and DMB) were present in the crystal lattice.

Crystals of *Mj*CobT in complex with nicotinic acid mononucleotide (NaMN) and nicotinic acid with α-ribazole 5′-phosphate were obtained in a condition containing 100 mM MES/acetate buffer pH 5.5 and 25% PEG 1.5 K, with 1 mM DMB and 25 mM Na/K-PO_4_ pH 7.0 added to the protein. Crystals were soaked in a 20% ethylene glycol, 100 MES/acetate buffer pH 5.5, 16% PEG 4 K, 1 mM nicotinic acid mononucleotide (NaMN) solution overnight prior to freezing.

Crystals of *Mj*CobT in complex with α-AMP and nicotinic acid were grown in a condition containing 100 mM 4-(2-Hydroxyethyl)-1-piperazinepropanesulfonic acid (HEPPS) pH 8.5 and 22% PEG 8 K with both 1 mM adenine and 50 mM Na/K-PO_4_ pH 7 added to the protein. Before freezing, crystals were soaked for 30 min in 20% ethylene glycol, HEPPS pH 8.5, 22% PEG 8 K, and 25 mM Na/K-PO_4_ with substrates 1 mM NaMN and 1 mM adenine. The same substrates (α-AMP and nicotinic acid) were captured in the crystal lattice by similar means after soaking I crystals in a solution containing 30% PEG 8 K instead of 20% ethylene glycol and 20% PEG 8 K.

### X-ray data collection and processing

X-ray data were collected at the Advanced Photon Source and processed with XDS^30^, HKL-2000^31^, or HKL-3000^32^. Structures were determined by molecular replacement with the Phenix^33^ software package with the coordinates of the apo *Mj*CobT (RCSB accession number 3L0Z) as the search model. Model building and refinement were completed with Phenix^33^ CCP4, and COOT^34^ Model and refinement statistics are compiled in Table 1.

**Table 1:** X-ray data collection and model refinement statistics.

	Apo	NaMN and α-RP + nicotinic acid	α-AMP + nicotinic acid
X-ray source	19ID	19ID	19ID
Space group	P4_1_2_1_2	P2_1_	P2_1_
Subunits per asymmetric unit	1	2	2
Cell dimensions (Å, °)	56.1, 56.1, 243.7	47.9, 139.09, 54.490 β = 111.67	46.4, 138.6, 51.2 β = 108.32
Resolution limits (Å)	37.7—2.15 (2.23–2.15)^a^	40.93–2.293 (2.375–2.293)	48.56–1.40 (1.45–1.40)
Number of independent reflections	22,116 (2113)	28,757 (2758)	118,692 (11,857)
Completeness (%)	99.6 (98.6)	97.39 (92.79)	98.9 (99.0)
Redundancy	12.4 (12.2)	6.9 (6.9)	6.8 (6.8)
avg I/avg σ(I)	28.4 (3.15)	15.50 (2.53)	12.2 (2.92)
*R*_merge_ (%)^b^	5.48 (69.2)	6.46 (47.7)	9.63 (66.7)
*R*-factor^c^ (overall)/no. reflections	0.207/22,069	0.196/28,677	15.4/118,682
*R*-factor (working)/no. reflections	0.205 (0.273)/20,077	0.218 (0.029)/27,244	0.153/113,418
*R*-factor (free)no. reflections	0.245 (0.325)/1030	0.210 (0.390/1433	0.175/5933
Number of protein atoms	2593	4386	5264
**Average B values**			
Protein atoms (Å^2^)	63.6	65.9	16.1
Ligand (Å^2^)	58.47	68.9	12.1
**Weighted RMS deviations from ideality**			
Bond lengths (Å)	0.008	0.014	0.015
Bond angles (°)	1.01	1.62	1.89
Planar groups (Å)	0.006		
**Ramachandran regions** (%)^d^			
Most favored	93.2	92.27	97.5
Additionally allowed	5.9	7.43	2.3
Generously allowed	0.9	0.29	0.3
**RCSB accession codes**	6PTF	6PU6	6PT8

^a^Statistics for the highest resolution bin.

^b^*R*_sym_ = (∑|I − *I*|/∑ I) × 100.

^c^*R*-factor = (Σ||*F*_o_| − I*F*_c_||/Σ|*F*_o_|) where |*F*_o_| is the observed structure-factor amplitude and |*F*_c_| is the calculated structure-factor amplitude.

^d^Distribution of Ramachandran angles according to PROCHECK^35^.

## Data Availability

All the data generated by this work is reported in the paper and coordinates for the reported structures have been submitted to the RCSB PDB. PDB codes are reported in the paper. X-ray coordinates have been deposited in the Research Collaboratory or Structural Bioinformatics, Rutgers University, New Brunswick NJ (accession nos. 6PT8, 6PU6, and 6PTF).
